# Honey-Based Mixtures Used in Home Medicine by Nonindigenous Population of Misiones, Argentina

**DOI:** 10.1155/2012/579350

**Published:** 2012-01-22

**Authors:** Monika Kujawska, Fernando Zamudio, Norma I. Hilgert

**Affiliations:** ^1^Instituto de Biología Subtropical, Facultad de Ciencias Forestales, Universidad Nacional de Misiones, Andresito 21, Misiones, 3370 Puerto Iguazú, Argentina; ^2^Department of Ethnology and Cultural Anthropology, University of Wroclaw, Szewska 50/51, 50-139 Wrocław, Poland; ^3^Consejo Nacional de Investigaciones Científicas y Técnicas, CONICET, Argentina

## Abstract

Honey-based mixtures used in home medicine by nonindigenous population of Misiones, Argentina. Medicinal mixtures are an underinvestigated issue in ethnomedical literature concerning Misiones, one of the most bioculturally diverse province of Argentina. The new culturally sensitive politics of the Provincial Health System is a response to cultural practices based on the medicinal use of plant and animal products in the home medicine of the local population. Honey-based medicinal formulas were investigated through interviews with 39 farmers of mixed cultural (*Criollos*) and Polish origins in northern Misiones. Fifty plant species and 8 animal products are employed in honey-based medicines. Plants are the most dominant and variable elements of mixtures. Most of the mixtures are food medicines. The role of honey in more than 90% of formulas is perceived as therapeutic. The ecological distribution of taxa and the cultural aspects of mixtures are discussed, particularly the European and American influences that have shaped the character of multispecies medicinal recipes.

## 1. Introduction

The province of Misiones in Argentina displays a complex biocultural mosaic. The original inhabitants of this territory are Mby'a Guaraní indigenous people, who nowadays live in reserves and in rural areas in neighborhoods with nonindigenous farmers [1, 2]. In the 20th century Misiones received a massive wave of transnational migration from nearly all European countries, a few Asian countries, and neighboring Paraguay and Brazil, as well as internal migrants from other Argentinean regions [3, 4].

Misiones harbors one of the richest plant biodiversity in the whole Argentinean territory [5]. This is reflected in the number of plant species recognized as medicinal resources. Over 300 medicinal botanical taxa have been documented in ethnobotanical and ethnopharmacological works from this region [6–9]. The use of plants for health care is an important practice for Mby'a aboriginal people, as well as Mestizo and European migrants living there. They are employed in home medicine by lay persons and used by healing specialists (called *payé*, *curandero*, and *médico*) [10–12].

Medicinal plants not only are relevant resources as part of valuable traditional lore, but also play an essential role in the ethnomedical systems of people who have no health insurance. In Andresito municipality, located close to our study area, 77 percent of the population has no health insurance [9]. These people can only use hospitals and Primary Health Care Centers—sort of basic ambulatories—free of charge. On the other hand, easy access to Primary Health Care is one of the priorities for the local government in Misiones. In the period 2004–2007, according to the Federal Health Plan, 1000 new health promoters and 90 community doctors were incorporated into the Primary Health System [9]. In the same period, a few phytomedicines based on native plants (*Maytenus ilicifolia* Mart. ex Reissek, *Cecropia pachystachya* Trècul.) and well-appreciated exotic species (*Calendula officinalis* L.) were introduced into the market and distributed for free in Primary Health Care Centers [13, 14]. The aforementioned plants form part of popular pharmacopoeia [1, 7–9]. This fact illustrates the new culturally sensitive approach of the National/Provincial Health System, as well as an effort to make remedies more economically accessible. A new objective of the Provincial Ministry of Health is to provide training for health personnel and to inform farmers and the general population about rational and responsible attitudes towards the use of medicinal plants [9]. To achieve this goal, further investigation on knowledge and practices related to medicinal resource is needed. To date, complementary medicine, including phytotherapy, has been sparsely undertaken as a research subject by investigators in Misiones, in comparison to the biocultural diversity of the province. Moreover, some researchers, who studied the medicinal use of plants by aboriginal communities of Mby'a, have pointed out that these people have quite an individualistic approach towards plant selection and the choice of certain species [1, 11]. This fact makes local phytotherapy even more complex. 

Honey is an important alimentary and medicinal resource in the region [15, 16]. A large number of wild species produce honey from the order Hymenoptera (bees, bumblebees, and wasps). The use of honey from stingless bees is a cultural marker of identity for the original inhabitants of Misiones—Guaraní communities, for whom these bees (Meliponini tribe) play an important role in the cosmology [17].

We still know very little about the diversity of uses of honey among the inhabitants of Misiones and South America, in general. Few studies have focused on honey-based remedies and they represent isolated pieces of information within the wide scope of ethnobotanical studies [18–22]. Therefore, only vague conclusions can be generated about the specificity of honey-based medicines and about the role of honey in multispecies formulas.

Our research among *Criollos* (people of mixed Amerindian and European origin) and Polish migrants had a pioneering and preliminary character [16]. It shows that *Criollos* and Polish migrants recognize and use the honey of 17 different ethnospecies, 7 of which have been employed in the treatment of a variety of ailments and illnesses. Honey is also applied as food medicine in health promotion and prophylactics. According to the local aesthetics, it is highly desirable to have a nest of stingless bees on the household premises. It is believed that the presence of bees brings prosperity to the household [16]. Therefore, honey and bees, specially stingless bee species, are very positively associated, which should favor their use and conservation. Both for *Criollos* and Polish settlers the most culturally important species are the native *yateí* (*Tetragonisca fiebrigi*) and the European honey bee—abeja—(*Apis mellifera*) [16].

The use of plants and animal products in home medicine forms a part of traditional ecological knowledge, which implies a close relationship between people and places [23]. Research among aboriginal and *Criollos* communities indicates the important relationship they have with forested areas as a source of natural medicines. Mby'a Guaraní maintain tight and strong bonds with the forest as the principal source of their existence and the frame of all their symbolic constructions [2]. Moreau [9] documented the use of 112 noncultivated medicinal plants among *Criollos* and migrants from Brazil in the north of Misiones. These people use plants from all habitats, however, primary and secondary forests provide the greatest number of medicinal species for them, followed by ruderal areas. Keller and Romero [8] in their study among *Criollos* farmers in the center of Misiones found that the majority of 176 botanical taxa employed in home medicine were noncultivated species. Forty-four percent of the species were cultivated in home gardens and fields, and only 3 percent were purchased in the market. There are no publications reporting on the importance of habitat for Polish or other European migrants in the context of medicinal resource use. According to Polish folk pharmacopoeia, Polish peasants have employed predominantly noncultivated herbaceous species, which grew in their home gardens, fields, and on pathway edges [24–26].

Plant and animal mixtures employed in the home medicine of local societies are understudied in ethnobiological and ethnopharmacological literature [27–29]. To date, just one piece of ethnobiological research has treated multispecies formulas used in the home medicine of the inhabitants of Misiones [16]. With this contribution, we would like to complement our previous publication. To do so we address and compare (1) the importance of plants and animal products employed in honey-based medicinal mixtures, (2) the role of honey in multispecies formulas, (3) ailments treated with these resources, and (4) source areas and forms of obtaining medicinal resources.

We hypothesize that different ethnospecies of honey bees and medicinal plants have different cultural and therapeutic importance for *Criollos* and Polish farmers. These groups explore different habitats for the procurement of medicinal species.

## 2. Material and Methods

### 2.1. Study Area

The research was conducted in the northern part of Misiones, which forms a part of the Atlantic Forest Ecoregion [30]. This is a semideciduous forest growing in a subtropical climate with hot summers (35–40°C), between December and March, and winters with frosts between June and August. Average annual rainfall is 1700–2200 mm, with no marked dry season [31].

The most important economic activities in the region are forestry and agriculture, supplemented by cattle breeding. The first is based on monoculture plantations of exotic species of pine (*Pinus* spp.) and eucalyptus (*Eucalyptus* spp.) for the paper and timber industry. The main crops are tobacco (*Nicotiana tabacum*), yerba mate (*Ilex paraguariensis*), té (*Thea sinensis*), and fruit plantations, mainly citrus. The provincial economy is based on raw material extraction with little industrial development [32, 33].

**Figure 1:**
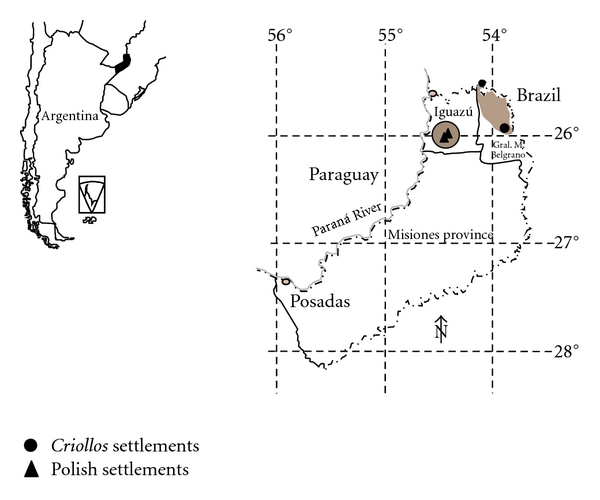
Study area.

The research was conducted in two departments: Iguazú and General Manuel Belgrano. Both of them share a border with Brazil (in the north and northeast, resp.). The department of Iguazú is also bordered by Paraguay in the west. We worked in two areas: Wanda and Gobernador J. J. Lanusse settlements, in the department of Iguazú, and in hamlets in the Andresito Península and Maria Soledad municipality, Andresito Guaçurarí, and San Antonio (Figure 1).

Wanda and Lanusse were established as Polish rural settlements. Wanda, situated on the banks of the Parana River, developed into a town with semirural lots on its outskirts. The Lanusse locality, 36 km from Wanda, conserved its rural character with a poor infrastructure. Nowadays Wanda and Lanusse exhibit a multicultural character: in addition to the Polish pioneers and their children, Argentinean migrants from other provinces as well as Paraguayan and Brazilian immigrants live there.

The study sites of Peninsula Andresito and Maria Soledad (department of General Manuel Belgrano) are hamlets with minimal infrastructure (dusty unpaved roads, not completely electrified areas, satellite schools). They are inhabited by small farmers of mixed cultural backgrounds (hereafter called *Criollos*). Some people obtained their lots through a settlement scheme, which dates back to the 1980s and by spontaneous occupation that was subsequent to the foundation of the overseas immigrant colonies [32, 33]. Among the inhabitants of the study area are Argentineans and Brazilians, descendants of Germans and other European people who live neighboring the Paraguayan and Argentinean Mestizos. The distinction between Polish and *Criollos *communities rests on three criteria: self-identification, language and customs, and cultural institutions (see Zamudio et al. [16]).

### 2.2. Data Gathering

This field work took place in 2009. After obtaining prior informed consent, an open-ended questionnaire was applied in the Spanish language. It consisted of seven sections with questions that sought to understand the contexts of use of the honey of Hymenoptera. With regard to the medicinal uses assigned to honey and other products derived from it, we aimed to investigate (a) the illnesses treated, (b) the forms of preparation and administration of remedies that include honey, and (c) the role of honey in the preparation of home remedies when they included other elements (plants, animal products, pharmaceuticals). Additional questions were asked about the place and mode of obtaining the mentioned resources (honey and plants). Illnesses are reported according to local ethnomedical terminology and classification. Only the reports of self-experience were included in the analysis, thus reports of uses not tried by the study participants were discarded.

We worked with 16 Polish settlers and their descendants (hereafter called Poles or Polish migrants), mainly from the first and, occasionally, second generation born in Argentina, aged between 39 and 77 (9 men and 7 women), and with 23 *Criollos* farmers, aged between 20 and 70 (14 men and 9 women). In both cases, the proportion between men and women was similar. We worked with all the families that we found present at home at the time of our arrival, except if they were close relatives of one already visited. The inclusion criteria were: age—only adults were interviewed—and cultural background; we tried not to include descendants whose parents were mixed couples (Polish and *Criollos*). Only two participants from the Polish group have *Criollos* spouses. Almost all the interviewed farmers are small producers.

The plants were collected in the presence of the study participants. Then, voucher specimens were identified by the authors (Monika Kujawska) and deposited in the herbarium of the* Universidad Nacional del Noroeste* (UNNE). The herbarium references to the plant collection are provided in Zamudio et al. [16]. Some of the bees of the tribe Meliponini were identified by Claus Rasmussen (Department of Entomology, University of Illinois, USA) and Fernando Silveira (Department of Zoology, Federal University of Minas Gerais, Brazil), while the rest of the insects were identified by Fernando Zamudio. The insects were deposited in the entomological collection of the *Universidad Nacional de Misiones* (UNAM).

### 2.3. Data Analysis

In this analysis, we included bee species that were mentioned by at least three informants and referred only to the use of honey (not other products derived from bees). The relative importance (RI) was calculated for each plant and animal species based on the normalized number of pharmacological properties attributed to it and the number of body systems it treated [34]. Similarity between the Polish and *Criollos* population was evaluated for modes and places of obtaining the species by applying the Simpson Coefficient. This is a percentage index that uses presence-absence information and is interpreted as the proportion of shared taxa in relation to the site (the cultural group in our case) with fewer species. This particular index was chosen as it is appropriate for ethnobiological studies based on interviews, which yield variable data [35]. Parameters such as richness, shared and exclusive species are presented as well.

To evaluate the variability of the medicinal plants' use within each study group, the informants' consensus factor (ICF) was calculated [36]. To evaluate the variability of the medicinal resources' use between Polish and *Criollos*, the analysis of variance ANOVA was performed using consensus factor data transformed by the logarithm Log 10. The Infostat program was applied for these analyses [37]. Four categories were defined as sources of medicinal plants and animal products: home units, transformed habitats, forests, and markets. Home units comprise house premises including front- and backyard, fenced home gardens adjacent to the house, and orchards. Transformed habitats are environments where productive activities take place: cultivated fields, pasture (for cattle breeding) and also highly modified ruderal areas—roadsides and ecotone between farm and forest. Forest areas embrace disturbed primary and secondary forests, which retain the basic tree layer vegetation characteristic of the study area. This category includes also natural wetlands found in lower areas of the forest and in coastal environments adjacent to river courses.

## 3. Results

### 3.1. Medicinal Resources

We registered the use of 58 different taxa in the course of the field research. Fifty of them are botanical taxa (including 5 industrially processed plant products), belonging to 38 genera and 27 botanical families. Eight taxa are products of animal origin: honey of 5 bee species and fatty products extracted from three vertebrates (Table 1). *Criollos* mentioned 45, while Polish migrants 33 taxa. Twenty-seven taxa were mentioned exclusively by *Criollos *and 14 by Poles. The study groups share the knowledge and the use of 18 plant and animal taxa, which constitutes nearly one-third of all mentioned medicinal resources.

The study participants reported 109 medicinal uses, which included a single-species remedy—pure honey—and 214 uses in which honey was combined with other elements: with one plant species (39.3%), with two or more plant species (16.4%), with vegetable oil (5.6%), with milk (5.1%), with animal fat (4.2%), with two or more plants and a pharmaceutical (3.7%), with one plant and vegetable oil (2.8%), with one plant and a pharmaceutical (2.8%), with one plant and alcoholic beverage (2.3%), with alcoholic beverages (1.9%), with at least two plants and vegetable oil (0.9%), and with a pharmaceutical, and with one plan and fat (0.5%) each (Tables 1 and 2).

The single-species remedies refer to honey of 5 bee taxa: European honey bee—abeja—(*Apis mellifera*), yateí (*Tetragonisca fiebrigi*), mandasaia (cfr. *Melipona quadrifasciata*), mirí (*Plebeia *spp.), and carabozá (*Trigona spinipes*). Abeja and yateí have the highest relative importance value. Pure honey is used both internally and externally to treat, in the first place respiratory system disorders (Poles: 67.3%, *Criollos* 54%). However, the order of importance changes between groups for other body systems. Poles use pure honey to treat ailments of digestive system (10.9%), ophthalmic (9.1%), skin and humoral medicine syndromes (3.6%), circulatory, and psychological and general problems (3.6%). In contrast, *Criollos* use pure honey to treat skin (16%), digestive, ophthalmic, and musculoskeletal (8%), psychological problems (4%), and general (2%).

During the interviews, the participants were asked, which components in the mentioned mixtures had medicinal properties for them, and whether honey could be replaced by sugar. By this question, we aimed to determine the role of honey in multispecies formulas: as a medicine or as a sweetener. Over 90 percent of *Criollos* respondents considered all products as necessary components of mixtures, either containing abeja or yateí honey (as only these species were used in mixtures). Phrases like “both of them make a remedy” or “if you join them, then the medicine becomes more powerful” were common responses. Only in one case was honey employed as sweetener, in a mixture with vegetable oil for pediatric use. In another five cases *Criollos* informants stated that if they did not have honey, they used burned sugar, but honey was better than sugar. Respondents also remarked that if they did not have a plant or animal product, which they used normally, they replaced it with another element or prepared a remedy without it. For example, on one occasion an informant said that a mixture of ajo (*A. sativum*) and oil was also effective against flu, if there was no honey to be added, but they conceived this mixture to be less powerful. Seventy-two percent of Polish participants stated that all the components in mixtures had medicinal properties. The lower percentage provided by Poles in comparison with *Criollos *may be due to the fact that Polish migrants mentioned more mixtures with tea and milk than *Criollos*, and these products were not always considered as medicinal resources but rather as conveyance mediums for proper medicine (honey or a plant). Both Poles and *Criollos *stressed that it was important to drink something hot when one was ill to make the body sweat, especially in the case of respiratory illnesses. In four cases Poles mentioned that honey could be replaced by sugar and its role was to make the following mixtures more palatable: (1) mixture with capybara fat; (2) three cases of multispecies formulas containing bitter plants, which were prepared for children.

**Figure 2:**
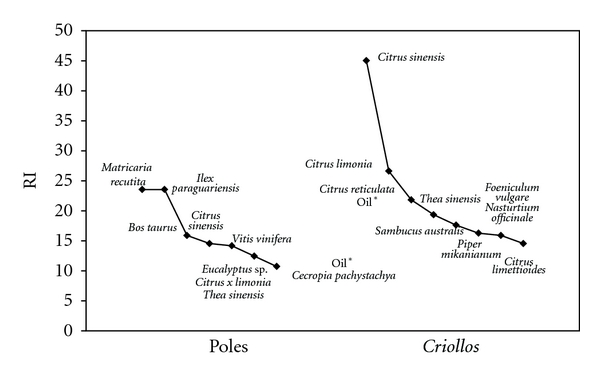
Rank-abundance curves (adapted) of species with the ten highest relative importance (RI) values by study group.

The relative importance (RI) analysis showed that different species are important for each of the study groups (Figure 2). The two most important species (excluding honey) for Poles are manzanilla (*M. recutita* L.) and yerba mate (*I. paraguariensis* A. St.-Hil.). None of these plants were mentioned by *Criollos*. The third most versatile product for Polish migrants, cow's milk (*Bos taurus*), was reported by *Criollos* only once. Among the ten most important medicinal resources used by *Criollos*, 6 were not mentioned even once by Poles. The exclusive species are native (*Sambucus australis* Cham & Schltdl. and *Piper mikanianum *(Kunth) Steud.) and adventitious plants (*Citrus sinensis *(L.) Osbeck, *C. reticulata *Blanco, *Foeniculum vulgare *Mill., *Nasturtium officinale*  R. Br.). Medicinal resources used by *Criollos *achieved in general a higher RI value that those used by Poles. This indicates that medicinal plants, especially citrus, have more versatile applications in this community, hence they are used to treat a larger number of body systems, and a greater number of pharmacological properties is attributed to them.

### 3.2. The Conception of Environment in Relation to Medicinal Resources

**Figure 3:**
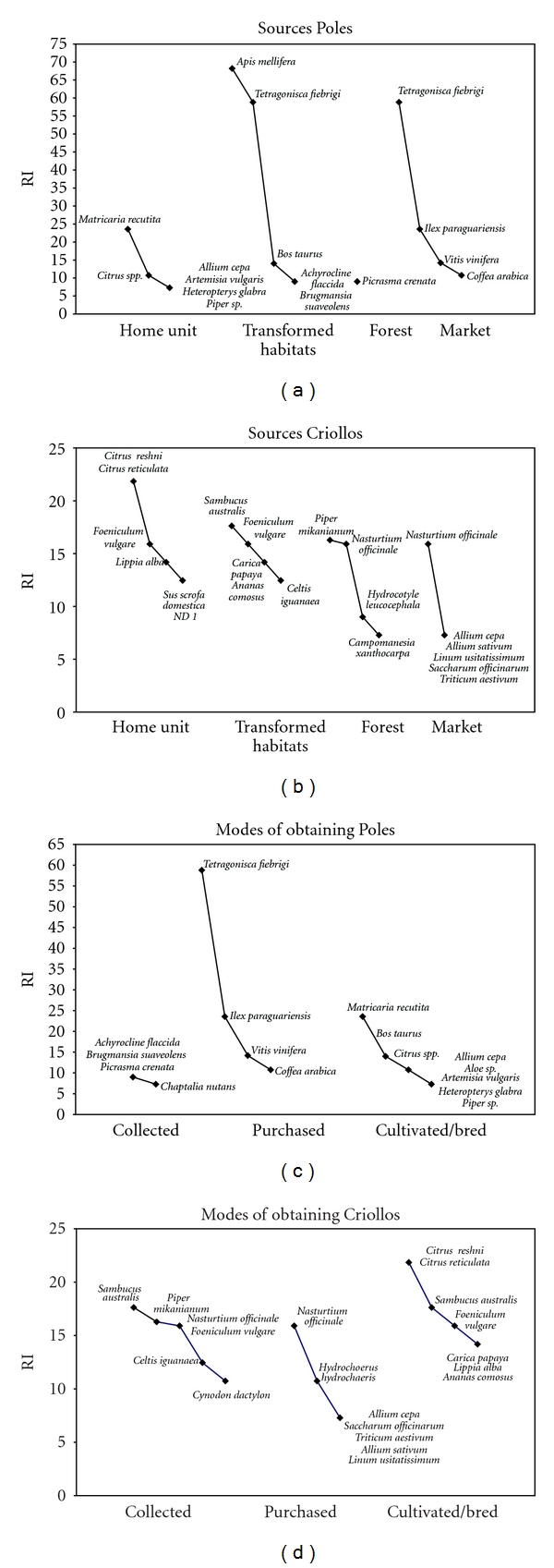
Rank-abundance curves (adapted) of exclusive species with higher relative importance (RI) value according to source and mode of obtaining resources by Poles and *Criollos* in honey-based mixtures.

The taxa used by Polish migrants and *Criollos* come from the home unit in the first place (43.8 and 45.7%, resp.) and from local markets in the second place (31.3 and 26.1%, resp.) (Table 3). The species originating from transformed habitats, which include fields, pastures, and pathways, present the lowest similarity between Poles and *Criollos*, with two shared species (Table 3 and Figure 3). By contrast, species obtained from the forest have achieved the greatest similarity (Table 3). This means that species from the forest are better defined for both groups, while there is great variability in selection of resources from places like gardens, pathways, cultivated fields, and pastures.

More than 50 percent of the taxa are cultivated or bred in both groups, but the similarity of cultivated and bred taxa between *Criollos *and Poles is the lowest (Table 3). On the other hand, the purchased and collected medicinal resources show the highest similarity between these two groups, although in no case did the similarity exceed 60 percent. Generally, these are species with low RI value, with the exception of bee and citrus species reported by both groups. Bee species (*T. fiebrigi* and *A. mellifera*), ajo (*A. sativum*), milk (*Bos taurus*), and the fat of capybara (*Hydrochaerus hydrochaeris*) are shared by both groups but are obtained from different places and through different modes, which leads to exclusivity. For example, Poles obtain yateí's honey (*T. fiebrigi*) from hives kept in transformed habitats (farms) or purchase it in the market, and *Criollos* breed this species on the house premises or obtain the yateí's honey from the forest. Even within the same group, resources are obtained from two or three different places, which is the case of capybara's fat (*Hydrochaerus hydrochaeris*). *Criollos* hunt this animal in the forest or purchase it from informal posts or local markets.

### 3.3. Variability in the Ailments' Treatment and Local Pharmacopoeia

The registered medicinal resources are employed in the treatment of diverse ailments, which were lumped into 9 body system disorders. There is a total consensus among both groups and within each community in the treatment of ophthalmologic problems, and a high consensus in the respiratory symptoms and illnesses (0.86, 0.88 Polish and *Criollos,* resp.). Additionally, *Criollos* agree on how to treat skin problems (077). Within the Polish migrant community, there is a medium consensus with respect to treatment of skin, gastrointestinal, and general problems (Table 4). Statistical test did not show any significant differences in the consensus about the species used in the treatment of the different corporal system between the study groups (*F* = 0.33, *P* = 0.726; *α* = 0.05).

## 4. Discussion and Conclusions

Working with an ethnobiological questionnaire on the use of honey in nonindigenous communities of northern Misiones led us to discuss medicinal plants. The presented results illustrate the rich complexity of home medicine among *Criollos* and Polish migrants [8, 9, 12]. A low diversity in the medicinal use of animal products in home therapies contrasts with the high number of plant resources employed in multispecies formulas. The prevalence of botanical resources in comparison with animal products documented in our research reflects a general trend in the local pharmacopoeias of other ethnic groups in Argentina [38–42]. The relative absence of honey in local pharmacopoeias may be explained by the narrow perspectives taken by researchers, who aim to address a given problem deeply, which inevitably leads to the partition of cultural domains into unnatural categories. Thus, honey is poorly reported as a medicinal resource in both ethnozoological and ethnobotanical studies.

The botanical species mentioned by study participants include groups of plants of different cultural origins, as a result of the cultural blending that took place in Misiones in the 20th century. A relatively high number of mentioned taxa are used exclusively by each of the study groups, although they live in the same area (60 km apart), which indicates a great local heterogeneity in choices of medicinal resources. This can be explained by the continuity/discontinuity of medical traditions and the cultural influences of the neighboring countries, which affect Polish migrants and *Criollos* differently, according to their geographical proximity.

The rich tradition of using medicinal plants and the honey of stingless bees by *Criollos* is explained by their longer presence in the region and the influence of both indigenous knowledge and European ethnomedical heritage [16]. Polish migrants, culturally more homogeneus than *Criollos*, could not continue their phytotherapy undisturbed in the subtropics of Argentina, due to the difference in local flora. However, they partly maintained their traditional herbal pharmacopoeia thanks to medicinal plant species, which they brought and found in Misiones (e.g., *M. recutita* L., *Arthemisia absinthium* L.), and globalized food plant species, mainly vegetables, which they found in Misiones and continued to use in their home therapies [12]. We presume that Polish migrants are mostly influenced by Paraguayan Mestizo culture in their use of natural medicinal resources. Some of the species used by Poles, for example, lengua de vaca (*Chaptalia nutans* (L.) Pol.), which is applied topically together with honey to treat injuries, has not been registered in any of the research among *Criollos* in Misiones, but has been documented in Paraguayan ethnobotanical literature [43]. On the other hand, *Criollos* from our research are more influenced by Brazilian culture, which is reflected in plant and animal nomenclature [44]. 

The most important plant species—with the highest RI value for *Criollos*—are citrus species, nonnative to the area, coinciding with the *Criollos* populations of Yungas [40]. Citrus was introduced by Jesuit monks in colonial times and are now cultivated as cash crops in Misiones [45–47]. The botanical species with the highest RI values used by Polish migrants reflect a blend of Polish and local traditions: manzanilla (*M. recutita*), which is the most important medicinal plant in Polish traditional pharmacopoeia [24] on the one hand, and on the other hand, yerba mate (*I. paraguariensis*), which is a native species, the base for the most popular social drink in this province [9]. It is a species that both *Criollos* and Polish migrants use daily, but according to our research, they use it differently. Poles employ it in a mixture with honey as food medicine to treat and prevent illnesses and *Criollos* use it as a social drink, which sometimes serves as a conveyance medium in order to ingest medicinal plants, which are added to it, but not with honey [9].

### 4.1. Multispecies Medicinal Formulas

The use of honey in medicinal mixtures has a long tradition in America [18]. In our research the employment of honey in mixtures prevails over the use of pure honey, in contrast to the study of Modro and colleagues in Brazil [22]. Plants are the most numerous resources in honey based multispecies formulas in both study groups. At the same time, plants and plant products are the most variable ingredients in these formulas. While *Criollos* employ more plants in mixtures than Polish migrants, the latter combine significantly more fatty products like milk, lard, and vegetable oil together with honey. Nevertheless, *Criollos'* multispecies medicines are significantly more complex: on average they mix 2.83 species, and Poles 1.63 species [16]. To the best of our knowledge, the analysis of single- versus multispecies medicines has not been undertaken as a research problem in Misiones. Therefore, it is difficult to claim that *Criollos* prefer mixtures over single-species medicines, although our research may indicate so in the addressed domain. Some suggestions also provide compilations on medicinal plants in other parts of Atlantic Forest, which include a great number of herbal mixtures [48]. In Polish folk pharmacopoeia, there is a strong prevalence of single-species remedies in comparison with medicinal mixtures. If applied, multispecies formulas have a low-component character [24, 26, 49]. Therefore, Polish migrants may have conserved this feature of their traditional phytotherapy, which was strengthened in Misiones, in the face of new experience with unknown flora.

Other studies on medicinal mixtures conducted in Latin America document their low-component character in home therapies. Lay people prepare mixtures predominantly made of 2-3 species at home, which is much fewer than those prepared by professional healers [27, 29, 40]. This fact is explained by the ingredients' availability, the time needed to prepare complex medicines [27] as well as the specialized knowledge of professional healers [40].

This research shows that the availability of products partly determines the composition of mixtures. If one component is not available, then the medicine is still prepared without it, or a missing ingredient is replaced by something else. Different species of citrus are especially subject to easy substitution. Therefore, there is a certain flexibility in the composition of medicinal formulas used by *Criollos* and Poles. The main purpose in combining different components is to make a medicine more effective, more “powerful.”

In the research conducted among the Dominicans, Vandebroek and colleagues [29] suggested that the infectious character and/or perceived seriousness of a health problem played a role in the choice of mixture instead of single-ingredient remedies. The main purpose of combining different herbal and nonherbal ingredients was to provide a guarantee that there would be a substance that would be effective in the treatment of a particular illness. Ford [50] described this as following the flexibility of northern Mexicans concerning medicinal plant use: *if one item is not available, because it is out of season, or climatic conditions are inappropriate, another can be used. If one item does not cure, an alternative can be tried, if one cure helps somewhat, two or three might be even better*. This could be an explanation in our area too, but it may be more likely that the described practices reflect an ample knowledge of different alternative uses of plants and animals apt for medicinal mixtures.

### 4.2. Honey-Based Mixtures as Food Medicines

Twenty-nine botanical species and 7 animal products (62 percent of the total) reported in our research are edible and are employed in the local diet [12, 51, 52]. Most of the registered mixtures are actually food medicines [53, 54]. The dominant character of mixtures as medicinal food is probably due to the dual function of honey as a medicinal and food resource among *Criollos* and Poles, who consume it on many occasions and in different contexts—as a tidbit and snack, as a component of the daily diet, and as a part of ceremonial food [16]. Additionally, food plants constitute an important part of local pharmacopoeias in the Atlantic Forest Ecoregion [55, 56].

The importance of food medicine has been well documented in Polish ethnomedicine [56]. Some of the formulas mentioned by migrants can be traced directly to Polish ethnomedical literature, for example, the blend of honey and milk used for coughs and sore throats [57]. *Criollos* use food plants originating from the Old World, like garlic—ajo—(*A. sativum* L.) and watercress—agrión—berro—(*Nasturtium officinale* R. Br.), which were not reported by Polish informants. In both groups edible herbal species are mainly nonnative vegetables and adventitious species. Also in other studies from Latin America there are many reports of the use of food plants in mixtures with honey, for example, different species of citrus, usually lemon [18, 19, 22, 27]. Cebolla (*A. cepa *L.) and ajo (*A. sativum*) are commonly used species in these preparations. The mixture of honey, onion, and oil was reported in Cuba [27], while honey and cebolla (*A. cepa*), and honey and ajo (*A. sativum*) are used medicinally in Brazil [19, 22]. Just as common is the use of vegetable oil, animal fat, and animal-derived products like milk and eggs. The mixture of fat (e.g., turtle or rhea fat) and honey is used to treat asthma, bronchitis, and flu in Brazil [21, 58, 59], Capybara oil (*Hydrochoerus hydrochaeris*), mentioned by both groups in our study, is one of the animals with medicinal properties most frequently cited by traders in the Federal District of Brazil [59]. Among the industrially processed plants, sugar (*Saccharum officinarum *L.) has been mentioned as a common ingredient especially in complex preparations such as bottles, syrups, and other mixtures [27, 29]. In contrast, in our research it is tea—té—(*T. sinensis *L.), which achieved the highest RI value and the highest frequency of mentions of all processed plants.

### 4.3. Ailments Treated with Honey-Based Medicines

Pure honey and honey-based mixtures are used primarily to treat respiratory symptoms and illnesses by both study groups. Additionally, there is a very high consensus within each group on the resources used in medicinal mixtures destined to treat respiratory system disorders. Among other body system disorders, skin problems are also commonly treated with honey-based medicines. However, *Criollos* use them more frequently than Poles do. Skin problems are treated predominantly with pure honey, or alternatively with a compress/poultice made from honey and a plant species. Honey is also considered a praised remedy for ophthalmic ailments. To treat eye problems, honey is applied exclusively in its pure form. Generally, the research shows that mixtures destined to treat internal problems are decisively more complex than those applied topically. Among other afflictions, digestive disorders and humoral symptoms gained some importance, especially among Poles. In the study region digestive disorders are treated predominantly with herbal medicines. Forty percent of all medicinal plants registered by Moreau [9] are used for digestive tract problems by the *Criollos* population. The results of our study are consistent with reports from other parts of Latin America. Honey, solely or in combination with other elements, is primarily employed in the treatment of respiratory system afflictions, followed by skin problems to a lesser extent, and with some variations in the order of importance, musculoskeletal, ophthalmic, circulatory, and digestive system disorders [19, 22, 27, 39]. Honey also has been reported as a tonic, an antidote to snake and dog bites, as an aphrodisiac, to treat culture-bound syndromes such as evil eye [19, 21, 39, 58, 60], and in mixtures to treat reproductive system disorders [39].

### 4.4. Medicinal Resources and Local Landscape

The selection of medicinal resources in home medicine is an ongoing process. In the study region, the pattern of plant and animal exploitation for medicinal purposes have been shaped by cultural and ecological aspects. As a consequence of the massive migration that Misiones witnessed, large portions of the Atlantic Forest of the Upper Parana have been converted into forest monocultures, cultivated fields and pastures. The nonindigenous population of the northern Misiones lives in modified and disturbed environments [61, 62].

Therefore, nearly all the species documented in this research come from modified habitats and home gardens. Very few of the species mentioned are collected from the primary forest, for example, *Picrasma crenata* (Vell.) Engl.; other plants, like *Bromelia balansae* Mez, *Campomanesia xanthocarpa* O.Berg, *Celtis iguanaea* (Jacq.) Sarg., *Hydrocotyle leucocephala* Cham. & Schltdl., and *Piper mikanianum* (Kunth) Steud. prosper both in primary and second growth forests. As for the honey of stingless bees, it is collected from the wild in the primary and secondary forest, but in the case of yateí and mirí, sometimes their nests are transported towards the household in logs, and honey is collected from there. The latter practice has been more often reported by Poles. Apart from ecological aspects, the dominant exploitation of home units by the study groups is due to the number of resources needed for complex remedies (see Cano and Volpato [27]). Keller and Romero [8] observed that *Criollos* from the central part of Misiones employed a similar number of cultivated and wild species in their home therapies, but the cultivated plants were used more frequently, due to easy access to them. The prevalence of plants and animal products originating from the home units is probably due to the same factor as in the case of *Criollos* from the center of Misiones—relatively small distance from medicinal resources. Strategies which aim to guarantee easy access to medicinal natural resources is a tendency observed in other parts of Atlantic Forest too, for example, among Caiçaras—farmers of mixed origin, and Afro-Brazilian communities living in the Atlantic Forest in the state of Santa Catarina, Brazil. These communities also explore managed and modified habitats in medicinal plant procurement [55, 62]. At the first sight, the practices of neighboring Mby'a communities, for whom the forest is the most important source of natural medicines, may contrast with the strategies of nonindigenous communities from the same region [2, 52]. Nevertheless, the Mby'a who camp or live in the forested areas also exploits the proximity of their households [1].

Over fifty percent of species and products used in honey-based mixtures are cultivated and nonnative to the area. The number of purchased and collected species is similar. The collected plants and animals are forest species and weeds, which prosper in ruderal and cultivated areas. There is a difference between Poles and *Criollos* in this respect. This is most probably due to the fact that *Criollos*, who have a longer presence in the area and are more influenced by indigenous communities, rely more on noncultivated species and explore more of the forest in search of plants and honey than Poles do. The relative importance of purchased products in medicinal formulas indicates that the knowledge of nonindigenous populations goes through a globalized process. The relevance of cultivated and purchased plant and animal products may be also due to the fact that honey-based mixtures are predominantly medicinal foods [16]. The low similarity, which we found between and within each of the study groups, considering the mode and place of obtaining medicinal resources is consistent with the diversity of approaches observed amongst Mby'a by Crivos and colleagues. Mby'a people as a cultural group share the same body of ecological knowledge, but individuals have different personal strategies for obtaining specific resources [1]. It is likely that the low similarity between Poles and *Criollos* is also due to cultural differences in their conceptions of the environment, and different approaches to management of environments.

This research has contributed to the documentation of local knowledge and practices concerning medicinal mixtures within the nonindigenous population of Misiones. On the one hand, we observe a great variability in honey-based mixtures, which reflects the inherited ethnomedical traditions of the study groups, a cultural legacy, which persisted throughout the migration process; then we observe the individualistic character of strategies and choices, as well as a heterogenic perception of resource availability between the cultural groups of Misiones. On the other hand, we observe a heterogeneity of practices within each cultural group. Heterogeneity in medicinal practices indicates variation in medicinal knowledge, which may positively influence knowledge exchange among individuals from the same community and between members of different cultural groups. It may also encourage people to experiment with plant and other medicinal resources.

On the whole, the new culturally sensitive politics of the Ministry of Health will make sense, if it shows a closer insight into this local dynamic and tries to understand what are the main social and environmental modelers involved in therapeutic choices. Therefore, new qualitative, in-depth approaches are needed to address the problem, such as stories of life, social networking, and knowledge exchange, so that future health actions are designed taking the appropriate local context into account [63–66].

Results like ours are also relevant to ethnopharmacological follow-up studies, because they inform how different elements are combined to create a desirable effect. Moreover, focusing solely on individual plants is not efficient in the treatment of those health afflictions that are preferentially treated with mixtures by local people (see Vandebroek et al. [29]).

## Figures and Tables

**Table 1:** Relative importance (RI) value of species used in honey-based mixtures by Criollos and Poles in north of Misiones, Argentina. P/C: Poles/Criollos, RIP: relative importance Poles, and RIC: relative importance *Criollos*.

Scientific name, botanical family (common name)	(#reports) P/C	RIP	RIC	Mode of preparation and administration	Ailment
*Achyrocline flaccida* (Weinm.) DC., Asteraceae (marcela)	2/0	9.00	—	Oral, infusion, with lemon, and abeja's honey, a pharmaceutical* (optatively)	Cough, influenza
*Allium cepa *L., Alliaceae (cebolla)	1/2	7.28	7.28	Oral, syrup, with abeja's honey	Catarrh
*Allium sativum* L., Alliaceae (ajo)	0/4	—	12.45	Oral, decoction, with eucalyptus, *guabirá*, oil, and abeja's honey	Bronchitis, pneumonia
				Oral, frying o mixed without cooking, with oil, and yateí's honey	Cough
*Aloe sp., *Xanthorrhoeaceae (aloe)	1/0	7.28	—	Oral, mixture, with abeja's honey	Tonsillitis
*Aloysia citriodora *Palau, Verbenaceae (cedrón)	0/2	—	9	Oral, Infusion, with orange, yateí's honey or burned sugar, and a pharmaceutical	Catarrh, cough
*Ananas comosus *(L.) Merr., Bromeliaceae (ananá)	0/11	—	12.45	Oral, syrup, abeja's honey	Influenza, respiratory tract, bronchitis, pneumonia, colds, cough
*Apis mellifera, *(abeja)	47/124	68.2	66.48	Oral, pure	Dyspepsia, constipation; nervous commotion; blood pressure imbalance, blood cleansing (humoral syndrome) preventive, influenza, colds, cough, sore throat, tonsillitis, bronchitis, pneumonia, catarrh
				Topic, pure	Injury, bruises, abscess; muscular pain
*Artemisia vulgaris *L., Asteraceae (sertal)	1/0	7.28	—	Oral, Infusion, with abeja's honey	Dyspepsia
*Bos taurus, *(leche)	12/1	14	7.28	Oral, mixture, with abeja or yateí's honey	Bronchitis, catarrh, influenza
(manteca)	1/0	9	—	Oral, mixture, with abeja's honey	Cough, sore throat
*Brassica oleracea *L., Brassicaceae (repollo)	0/2	—	7.28	Topic, Compress with yateí's honey	Bruises, abscess
*Bromelia balansae *Mez, Bromeliaceae (caraguatá)	0/1	—	7.28	Oral, syrup with abeja or yateí's honey	Influenza
*Brugmansia suaveolens *(Humb. & Bonpl. Ex Willd.) Bercht. & J. Presl, Solanaceae (floripón)	2/0	9.00	—	Topic, with yateí or mirí's honey	Injury
*Campomanesia xanthocarpa *O. Berg, Myrtaceae (guabirá)	0/1	—	7.28	Oral, decoction, with eucalyptus, garlic, oil, and abeja's honey	Bronchitis, pneumonia
*Carica papaya *L., Caricaceae (mamón)	0/18	—	14.18	Oral, decoction, with lemon, *ambay*, *berro*, *sauco*, *talera*, *salvia* with abeja or yateí's honey	Tonsillitis, catarrh, influenza
				Oral, syrup, *talera*, lemon, *pariparoba* with yateí's honey	Sore throat
*Cecropia pachystachya *Trécul, Cecropiaceae (ambay)	3/22	10.73	10.73	Oral, decoction, eucalyptus, citrus, and abeja's honey	Catarrh, sore throat, cough
				Oral, decoction, with lemon, *mamón*, *berro*, *sauco*,* talera*, *salvia* with abeja or yateí's honey	Tonsillitis, catarrh, influenza
				Oral, syrup with abeja or yateí's honey	Cough, bronchitis, pneumonia
*Celtis iguanaea *(Jacq.) Sarg., Celtidaceae (talera)	0/16	—	12.45	Oral, decoction, with lemon, *mamón*, *berro*, *sauco*, *talera*, salvia with abeja or yateí's honey	Tonsillitis, catarrh, influenza
				Oral, syrup, with *talera*, lemon, *pariparoba* with yateí's honey	Sore throat
*Cfr. Melipona quadrifasciata, *(mandasaia)	0/1	—	7.28	Oral, pure	Influenza
*Chaptalia nutans* (L.) Pol., Asteraceae (lengua de vaca)	1/0	7.28	—	Topic, compress, with carabozá, mirí o yateí's honey	Injury
*Citrus reshni *Hort ex Tan., Rutaceae (mandarina)	0/4	—	21.84	Oral, infusion, with abeja's honey	Preventive
*Citrus x limonia *(L.) Osbeck, Rutaceae (limón)	14/46	12.45	26.63	Oral, decoction, tea, abeja or yateí's honey. With red wine, a pharmaceutical (optatively)	Influenza, colds, sore throat, cough
				Oral, syrup, with orange and abeja's honey	Cough
				Oral, syrup, with yateí's honey	Respiratory tract
*Citrus aurantium *L., Rutaceae (apepú)	4/2	9	14.18	Oral, decoction, with tea, abeja or yateí' honey, a pharmaceutical (optatively)	Influenza, cough
				Oral, syrup, with abeja or yateí's honey	Influenza
*Citrus limettioides* Tanaka, Rutaceae (lima)	0/2	—	14.56	Oral, infusion, with abeja's honey	Cough, preventive
*Citrus limón *(L.) Osbeck, Rutaceae (limón)	ídem *Citrus x limonia *				
*Citrus paradisi *Macfad., Rutaceae (pomelo)	2/0	9.00	7.28	Oral, mixture, with abeja's honey	Influenza, cough
*Citrus reticulata *Blanco, Rutaceae (mandarina)	0/4	—	21.84	Oral, infusion, with abeja's honey	Preventive
*Citrus sinensis *(L.) Osbeck, Rutaceae (naranja)	2/20	14.56	45.02	Oral, syrup, with abeja or yateí's honey; or with lemon and abeja's honey; with *cedrón*, yateí's honey or burned sugar, a pharmaceutical (optatively)	Colds, cough, catarrh, respiratory tract, nervous commotion
				Topic, poultice, with abeja or yateí's honey	Bruises, abscess
*Citrus *spp., Rutaceae (cítrico)	3/0	10.73	—	Oral, decoction, with eucalyptus, *ambay*, abeja or yateí's honey	Sore throat, catarrh, cough, influenza
*Coffea arabica *L., Rubiaceae (café)	3/0	10.73	—	Oral, infusion, with lemon and abeja's honey; with abeja's honey	Influenza, colds; cough
*Cymbopogon citratus *(D.C.) Stapf, Poaceae (cedrón)	2/4	9	12.45	Oral, mixture o infusion, with oil and abeja's honey; infusion with orange, yateí's honey or burned sugar and a pharmaceutical	Cough, catarrh, bronchitis, pneumonia, influenza
*Cynodon dactylon *(L.) Pers., Poaceae (gramilla)	3	—	10.73	Oral, infusion, with yateí's honey	Fever
*Eucalyptus* sp., Myrtaceae (eucalipto)	4/6	12.45	9.00	Oral, decoction, with *ambay*, citrus, abeja, and yateí's honey	Sore throat, cough, catarrh
				Oral, syrup, with citrus and yateí's honey; decoction with* guabirá*, garlic, oil, and abeja's honey	Influenza
*Foeniculum vulgare *Mill., Apiaceae (aipo)	0/4	—	15.9	Oral, syrup, with *sabuguero* and abeja's honey	Respiratory tract
				Oral, infusion, with yateí's honey or burned sugar	Tonsillitis, sore throat
				Oral, decoction, with abeja's honey and a pharmaceutical	Colds
*Glycine max *(L.) Merr., Fabaceae (aceite vegetal)	3/13	10.73	21.46	Oral, mixture, with abeja or yateí's honey; decoction, with eucalyptus, *guabirá*, garlic, and abeja's honey	Sore throat, bronchitis, pneumonia, influenza
				Oral, mixture or infusion, with *cedrón* and abeja or yateí's honey; frying or mixture, with garlic and yateí's honey	Cough, catarrh
				Oral, mixture, with yateí's honey	Constipation
*Helianthus annuus *L., Asteraceae (aceite vegetal)	ídem *Glycine max *				
*Heteropterys glabra *Hook. Malpighiaceae (tilo)	1/0	7.28	—	Oral, infusion, with abeja's honey	Nervous commotion
*Hydrochoerus hydrochaeris, *(grasa de carpincho)	5/3	10.73	10.73	Oral, mixture, with abeja or yateí's honey; with tea and abeja's honey	Catarrh, respiratory tract
*Hydrocotyle leucocephala *Cham. & Schltdl., Apiaceae (oreja de gato)	0/2	—	9.00	Topic, poultice, with abeja or yateí's honey	Bruises, absecess
*Ilex paraguariensis *A. St.-Hill., Aquifoliaceae (yerba mate)	4/0	23.56	—	Oral, infusion, with abeja or yateí's honey	Blood pressure imbalance, nervous commotion, blood cleansing (humoral syndrome), to fortify the body
*Linum usitatissimum *L., Linaceae (lino)	0/1	—	7.28	Oral, infusion, with abeja or yateí's honey	Cough
*Lippia alba *(Mill.). N. E. Br., Verbenaceae (salvia)	0/11	—	14.18	Oral, decoction, with lemon, *ambay*, *berro*, *sauco*, *talera*, *mamón*, abeja or yateí's honey; with abeja's honey and a pharmaceuticals	Tonsillitis, catarrh, cough, influenza
*Matricaria recutita *L., Asteraceae (manzanilla)	4/0	23.56	—	Oral, infusion, with lemon and abeja or yateí's honey, a pharmaceutical (optatively)	Cough, sore throat, influenza, catarrh, colds, dyspepsia
*Mentha *spp., Lamiaceae (menta casera)	0/4	—	9.00	Oral, infusion, with abeja or yateí's honey	Colds, cough
*Nasturtium officinale *R. Br., Brassicaceae (agrión, berro)	0/13	—	15.90	Oral, decoction, with lemon, *ambay*, *salvia*, *sauco*, *talera*, *mamón*, with abeja or yateí's honey	Tonsillitis, catarrh, influenza
				Oral, syrup, with abeja's honey; with *guaco* and abeja's honey	Cough, influenza, bronchitis, pneumonia, asthma, tuberculosis; respiratory tract
Cfr.* Mikania* sp., Asteraceae (guaco)	0/5	—	12.45	Oral, syrup, with *guaco* and abeja's honey	Cough, influenza, bronchitis, pneumonia, asthma, tuberculosis
*Picrasma crenata *(Vell.) Engl., Simaroubaceae (palo amargo)	2/0	9.00	—	Oral, infusion, with abeja's honey	Dyspepsia
*Piper mikanianum* (Kunth) Steud., Piperaceae (pariparoba)	0/5	—	16.28	Oral, syrup, *talera*, lemon, *mamón*, yateí's honey	Sore throat
				Topic, compress, with abeja or yateí's honey	Injury
*Piper *sp., Piperaceae (matico)	1/0	7.28	—	Oral, infusion, with abeja's honey	Dyspepsia
*Plebeia *spp. (mirí)	9/0	23.18	16.28	Oral, pure	Influenza, tonsillitis, colds, asthma; bronchitis, pneumonia, cough, muscular pain
Saccharum officinarum L., Poaceae (azúcar quemada, burnt sugar)	0/6	—	7.28	Oral, infusion, with orange, *cedrón* and pharmaceutical; with *aipo *	Catarrh, cough catarrh, sore throat, cough
				Oral, decoction, eucalyptus, citrus, and *ambay *	Tonsillitis, sore throat
(aguardiente)				Oral, macerated with *sabuguero *	Asthma
*Sambucus australis *Cham. & Schltdl., Adoxaceae (sabuguero, sauco)	0/12	—	17.62	Oral, syrup, with *aipo* and abeja's honey	Respiratory tract
				Oral, macerated with aguardiente and abeja's honey	Asthma
*Sida cordifolia *L., Malvaceae (malva)	0/1	—	7.28	Oral, infusion, with bee or yateí's honey	Cough
*Sus scrofa domestica, *(grasa de chancho, pork fat)	0/4		12.45	Oral, mixture, with abeja or yateí's honey	Colds, bronchitis, pneumonia
*Tetragonisca fiebrigi, *(yateí)	37/68	59.20	58.81	Oral, pure	Cough, respiratory tract, catarrh, asthma, influenza, sore throat, bronchitis, pneumonia, asthma, thrush; nervous commotion, preventive, parasites; dyspepsia
				Topic, pure	Ocular illness; insect's bite; bruises, abscess, injury; bronchitis, pneumonia
*Thea sinensis *L., Theaceae (té común, té negro)	14/14	12.45	19.35	Oral, decoction, with lemon or *apepú* and abeja or yateí's honey, a pharmaceutical (optatively)	Influenza, colds, catarrh, cough
*Trigona spinipes, *(carabozá)	0/8	25.29	14.56	Oral, pure	Stomach fever (*fiebre de estómago*), influenza, tonsillitis, asthma
				Topic, pure	Rheumatic illness
*Triticum aestivum *L., Poaceae (trigo, harina)	0/3	—	7.28	Topic, poultice, with abeja or yateí's honey	Bruises, absecess
*Viola odorata *L., Violaceae (violeta)	0/2	—	7.28	Topic, poultice, with abeja or yateí's honey	Bruises, absecess
*Vitis vinifera *L., Vitaceae (vino tinto)	7/0	14.18	—	Oral, decoction, with lemon and abeja's honey; mixture, with abeja's honey	Colds, catarrh, cough, influenza
*Zea mays *L., Poaceae (aceite vegetal)	ídem *Glycine max *				

*Pharmaceuticals composed of acetylsalicylic acid, paracetamol, or ibuprofen.

**Table 2:** Numbers of affections according to body systems and forms of preparations.

Body system	no. affections	Forms of preparation
Respiratory	11	A (37; 27), B (18; 27), C (23; 23), D (7; 2), E (5; 6), F (10; 2), G (2; 0), H (0; 1), BD (0; 1), BE (4; 2), BG (5; 0), BH (2; 3); CE (0; 2); CH (1; 7)
Skin	5	A (1; 8), B (9; 12)
Humoral medicine	5	A (1; 0), B (4; 0), G (1; 0), BH (0; 1)
Digestive	4	A (6; 4), B (5; 0), E (0; 1)
Ophthalmic	1	A (5; 4)
Musculoskeletal	1	A (0; 4)
Psychological	1	A (1; 2), B (2; 1)
Circulatory	1	A (1; 0), B (1; 0)
General	1	A (1; 1), B (2; 1)

In parentheses no. reports (Poles; *Criollos*).

A (pure honey), B (honey, one plant species), C (honey, at least two plant species), D (honey, animal fat), E (honey, vegetable oil), F (honey, milk), G (honey, alcoholic beverage), H (honey, a pharmaceutical), BD (honey, a plant species, animal fat), BE (honey, a plant species, vegetable oil), BG (honey, a plant species, alcoholic beverage), BH (honey, a plant species, a pharmaceutical), CE (honey, at least two plant species, vegetable oil), and CH (honey, at least two plant species, a pharmaceutical).

**Table 3:** Species richness and similarity (Simpson Coefficient) between Poles and Criollos according to source and mode of obtaining resources used in honey-based mixtures.

	Source								Mode of obtaining					
	Home unit		Transformed habitats		Forest		Market		Collected		Purchased		Cultivated/bred	
Total species richness	27		16		10		16		18		17		34	
	Poles	*Criollos*	Poles	*Criollos*	Poles	*Criollos*	Poles	*Criollos*	Poles	*Criollos*	Poles	*Criollos*	Pole	*Criollos*
Richness	14	21	9	9	6	9	10	12	10	14	10	13	17	25
Relative %	43.8	45.7	28.1	19.6	18.8	19.6	31.3	26.1	31.3	30.4	31.3	28.3	53.1	54.3
Exclusive species	6	13	7	7	1	4	4	6	4	8	4	7	9	17
Shared species	8		2		5		6		6		6		8	
Simpson Coefficient	57.1		22.2		83.3		60		60		60		47.1	

**Table 4:** Informants' consensus factor, comparison between Poles and *Criollos* concerning medicinal plant use according to the body systems.

	Poles	*Criollos*
Respiratory	0.86	0.88
Skin	0.5	0.77
Humoral medicine	0.38	0
Digestive	0.64	0.43
Ophthalmic	1	1
Musculoskeletal	—	0.33
Psychological	0.33	0.33
Circulatory	0	—
General	0.5	0.14

*Factor_informants*'*  consensus_ = (*n*_use-reports_ − *n*_taxa_)/(*n*_use-reports_ − 1).

(A higher value indicates a high rate of agreement between the informants, a low one a low degree of agreement).
